# Front-Inner Lens for High Sensitivity of CMOS Image Sensors

**DOI:** 10.3390/s19071536

**Published:** 2019-03-29

**Authors:** Godeun Seok, Yunkyung Kim

**Affiliations:** Department of Electronics Engineering, Dong-A University, Busan 49315, Korea; wmf159tjr@gmail.com

**Keywords:** front-inner lens, CMOS image sensor, FDTD simulation

## Abstract

Due to the continuing improvements in camera technology, a high-resolution CMOS image sensor is required. However, a high-resolution camera requires that the pixel pitch is smaller than 1.0 μm in the limited sensor area. Accordingly, the optical performance of the pixel deteriorates with the aspect ratio. If the pixel depth is shallow, the aspect ratio is enhanced. Also, optical performance can improve if the sensitivity in the long wavelengths is guaranteed. In this current work, we propose a front-inner lens structure that enhances the sensitivity to the small pixel size and the shallow pixel depth. The front-inner lens was located on the front side of the backside illuminated pixel for enhancement of the absorption. The proposed structures in the 1.0 μm pixel pitch were investigated with 3D optical simulation. The pixel depths were 3.0, 2.0, and 1.0 μm. The materials of the front-inner lens were varied, including air and magnesium fluoride (MgF_2_). For analysis of the sensitivity enhancement, we compared the typical pixel with the suggested pixel and confirmed that the absorption rate of the suggested pixel was improved by a maximum of 7.27%, 10.47%, and 29.28% for 3.0, 2.0, and 1.0 μm pixel depths, respectively.

## 1. Introduction

Currently, high-resolution and high reality cameras are required for use in broadcasting, security, automotive, and various other systems. Moreover, 8K digital cameras or 8K video cameras for ultra-high-definition television (UHD TV) systems have been developed [1,2,3,4,5,6]. The International Telecommunication Union (ITU)-R has standardized video parameters for UHD TV as 7680 (H) × 4320 (V) pixels, 120 Hz frame frequency, etc. [1]. To meet these specifications, 8K or higher resolution complementary metal-oxide-semiconductor (CMOS) image sensors with high-speed and a high saturation signal have been developed [2,3,4,5,6]. Furthermore, the 8K resolution cameras can be adapted for mobile phones that have a sensor size limitation. The resolution of mobile phone cameras is also increasing with the demand for a highly functional camera. To achieve 8K or more of high resolution, a large number of pixels must exist in a limited chip area, so the pixel pitch has to shrink to less than 1.0 μm. Consequently, the aspect ratio gets worse, and the optical path gets longer and narrower. Therefore, the optical performance in sensitivity and crosstalk deteriorates. One solution to overcoming this problem is the reduction of pixel depth. A thin pixel makes the crosstalk lower and the camera module more compact. However, light with long wavelengths, such as with a red color, is absorbed in the deep pixel depth because the absorption depth is varied with each wavelength [7]. Also, the sensitivity is reduced by the shrinkage of the absorption volume. 

To enhance the sensitivity of the CMOS image sensor, a significant amount of research has been conducted [8,9,10,11,12,13,14,15,16,17]. In the pixel optical structure, the back side-illumination (BSI), deep trench isolation (DTI), vertical transfer gate (VTG), and the inner micro lens have been developed [8,9]. In the BSI structure, the photodiode is placed under the lens, and the wiring layer is placed under the photodiode [8]. Thus, sensitivity deterioration caused by the wiring layer can be reduced. However, the problem of crosstalk emerges with the reduction of pixel size. To overcome this problem, DTI and VTG structures were developed [9]. The DTI has a structure for preventing the interference between adjacent pixels by forming a barrier between pixels. However, the pixel volume, where the light is absorbed, becomes smaller by using the DTI. To solve this problem, VTG technology was applied to improve the sensitivity by changing the horizontal gate used for a typical BSI sensor. The sensitivity deterioration remains challenging, since the DTI cannot be applied for very small pixel areas under 1.0 μm. In addition to these structures, the optical elements such as the inner lens, the metal shield, the color filter (CF) separation wall, the lensed CF and the parabolic CF were introduced for high-sensitivity CMOS image sensors [10,11,12,13,14,15,16,17]. The inner lens, having a different refractive index with a photodiode is located above a photodiode to converge or diffuse the light received by the curve [10]. The problem with the inner lens is that it is difficult to stabilize because the incident light that must pass through the center of the lens is not at the center. Additionally, the metal shield is introduced for lowering the light leakage and crosstalk [11,12,13]. This metal shield is located above or below the CF [11,12], or is buried in the silicon [13]. However, crosstalk has not been reduced enough by the only metal shield. Also, the CF separation walls are inserted between adjacent color filters to reduce crosstalk [14,15]. However, separation walls cannot be adapted in a small pixel because CF width is also shrinking by separation walls. Additionally, the lensed CF, which unites the micro lens and a color filter in a single body, is proposed to reduce crosstalk and increase sensitivity without changing color filter materials [16]. Parabolic color filters have also been developed [17]. The optical efficiency increases when a micro lens and parabolic color filter are aligned with the same radius of curvature. However, the problem is that crosstalk gets worse when the radius of curvature increases. This research is difficult to adapt to commercial manufacturing. Despite all these studies, the problem of crosstalk and sensitivity deterioration still arises when the pixel pitch is below 1.0 μm. 

In this paper, we propose a highly sensitive pixel structure with a front-inner lens. This pixel structure exhibited improved sensitivity in the long wavelength, with 29.28% in the 1.0 μm pixel depths. The front-inner lens, located in the interconnection metal layer of the pixel, has a lower refractive index than the inter-dielectric layers. The shapes of the front-inner lens were investigated both in the hemisphere and in the square. To investigate optical performance, the absorbed photon density of the proposed structures in the 1.0 μm pixel pitch was simulated using a commercial 3D optical simulator. The pixel depths were 3.0, 2.0, and 1.0 μm for enhancement of the aspect ratio. The materials of the front-inner lens were varied using air and magnesium fluoride (MgF_2_). Section 2 describes the concept of the front-inner lens, Section 3 presents the simulation results and discusses the results, and conclusions are presented in Section 4.

## 2. Concept of the Front-Inner Lens

As previously mentioned, the aspect ratio gets worse with shrinking pixel size. Therefore, sensitivity decreases while the crosstalk increases due to the narrower and longer optical path. If the pixel depth is reduced to compensate for the aspect ratio, the crosstalk can be enhanced. However, the sensitivity gets worse, especially at long wavelengths such as in the red color spectrum. From the relation between the light’s absorption coefficient and the silicon as photodiode, the pixel depth must be at least 3.0 μm for long wavelengths to be absorbed [7]. To eliminate the optical loss in the long wavelengths, the front-inner lens structure is proposed when the pixel depth is less than 3.0 μm. As a result, the optical path becomes longer, even at a shallow pixel depth, and the sensitivity in the long wavelength is enhanced. 

**Figure 1:**
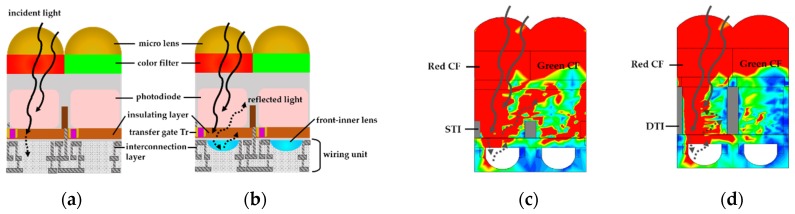
Pixel structures and beam profiles of power flux density. The incident light was a 650 nm wavelength. The solid line indicates the transmitted light from the micro lens and color filter. The dotted line shows the reflected light from the front-inner lens: (**a**) The incident light with the typical structure was not fully absorbed in the photodiode; (**b**) the incident light with the proposed structure was reflected and absorbed in the photodiode; (**c**) the proposed structure with shallow trench isolation (STI), and (**d**) the proposed structure with deep trench isolation (DTI).

In the case of the backside-illuminated pixel structure, the light enters the micro lens in the backside of the pixel [18]. The incident light transmits through the color filter and is absorbed in the photodiode. If the pixel depth is not sufficient, the light in the long wavelength cannot be absorbed and transmits the photodiode as shown in Figure 1a. The wiring (interconnection) unit for readout circuits is in the front side of the pixel. The insulating layer is located between the photodiode and the wiring unit, as shown in Figure 1a,b. The concept of the proposed structure is shown in Figure 1b. The front-inner lens is located in the front-side interconnection layer. Usually there is one interconnection line for the floating diffusion node in the insulating layer and the multi-layered interconnection metal line in the interconnection layer. For easy simulation, we assumed that there was only one interconnection metal layer of tungsten and no interconnecting metal line in the insulating layer. The front-inner lens, which has a lower refractive index than the inter-dielectric layer, caused the light of the long wavelength reflection and refraction to the photodiode. Therefore, the front-inner lens could reduce the absorption loss of long wavelengths. In this work, the materials of the front-inner lens were used by MgF_2_ and air for investigation of the proposed structure. Usually, the refractive index of the inter-dielectric layer was about 1.5 at a 650 nm wavelength [19]. The refractive indexes of MgF_2_ and air were 1.3767 and 1.0, respectively, at 650 nm wavelength [20]. When the refractive index of the front-inner lens was lower than the inter-dielectric layer, reflection occurred at the boundary of the front-inner lens. The reflection occurred again when the refracted light was passed to the front-inner lens. For the investigation of this reflection, the pixel depths were varied at 3.0, 2.0, and 1.0 μm. 

Contrastingly, the reflected light from the front-inner lens could be absorbed in the next photodiode as the crosstalk. The beam profile of the power flux density in the test simulation of the front-inner lens with air shows this phenomenon in Figure 1c,d. To show crosstalk well, the light was incident at 20°. The wavelength of the incident light was 650 nm. Simply put, the red region in the beam profile shows the transmitted light, which could be absorbed in the photodiode. In this work, the reduction of the crosstalk in the shallow trench isolation (STI) and DTI structures was compared as shown in Figure 1c,d. Figure 1c shows the light caused mostly by the reflection of the front-inner lens because the STI cannot block the crosstalk. On the contrary, the DTI can block the reflected light, as shown in Figure 1d. However, the DTI reduced the sensitivity due to the decrease in absorption because the area of the photodiode shrank, especially in the small pixel pitch. We simulate and compare STI and DTI in the following section. 

## 3. Simulation Results

For analysis of the proposed structure, we used a commercial 3D optical simulator with the finite-difference time-domain (FDTD) method [21]. The FDTD method has been established as useful for investigating the optical performances of CMOS image sensors [22,23]. The overall simulated conditions are shown in Table 1. The pixel pitch was fixed at 1.0 µm for the small pixel pitch. Pixel depths with thicknesses of 3.0, 2.0, and 1.0 µm were used to investigate the enhancement of the sensitivity by the front-inner lens at the thinner photodiode depth. The height and radius of curvature (ROC) of the micro lens were optimized for high sensitivity at 0.6 μm. The height and ROC of the front-inner lens were optimized at 0.25 and 0.3 μm, respectively. The wavelength of the incident light was 650 nm because the sensitivity in long wavelengths was affected by pixel depth. To investigate the sensitivity in the oblique incident light, the incident angles were set to 0°, 10°, and 20°. The refractive index of MgF_2_ and air were 1.3767 and 1.0 at 650 nm, respectively [20]. Pixels with tungsten grid (WG), STI, and DTI were investigated to enhance the crosstalk. STI is an element for separation between pixels. WG as a metal shield and DTI as a pixel isolation element are usually inserted in the pixel to reduce crosstalk. In this paper, an insulator—HfO2—was used for the STI and DTI. The width of WG was 0.075 μm and the height was 0.2 µm. The width of STI and DTI was 0.1 µm. The height of STI was 0.35 µm. The depths of DTI were 1.0, 2.0, and 3.0 µm depending on the depth of the pixel.

**Figure 2:**
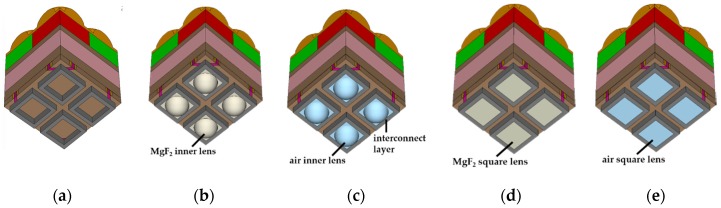
Simulated 3D pixel structures at the 1.0 μm pixel depth: (**a**) Typical structure, (**b**) front-inner lens with MgF_2_, (**c**) front-inner lens with air, (**d**) square front-inner lens with MgF_2_, and (**e**) square front-inner lens with air.

The simulated pixel structures in the case of pixel depth 1.0 µm are shown in Figure 2. The pixels of 2 × 2 arrays are shown on the front side because the front-inner lens was located in the front part of the pixel. As mentioned above, we assumed the interconnection metal layer was only one layer. For comparison, the typical structure has also been simulated in Figure 2a–c, and shows the simulated pixel structures with the spherical type of the front-inner lens. We also simulated the square type of the front-inner lens which could be processed easily, as shown in Figure 2d,e.

**Figure 3:**
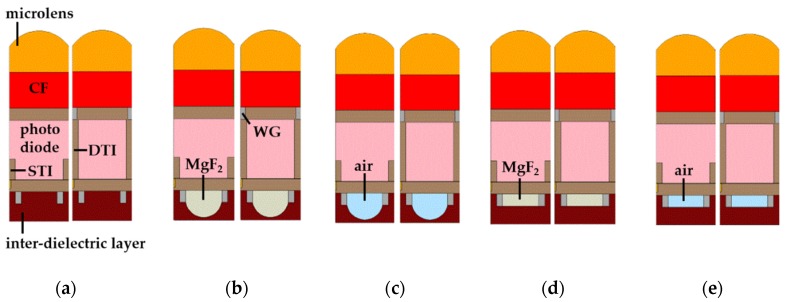
Cross-section of the simulated pixel structures in the 1.0 μm pixel depth. The left pixel of all cross-sections had STI, and the right pixel had DTI and WG: (**a**) Typical structure, (**b**) front-inner lens with MgF_2_, (**c**) front-inner lens with air, (**d**) square front-inner lens with MgF_2_, and (**e**) square front-inner lens with air.

Figure 3 shows the cross-sections of the simulated pixels at a pixel depth of 1.0 µm. There are two types of pixels: The left pixel had a STI, and the right pixel had a DTI with WG. 

**Figure 4:**
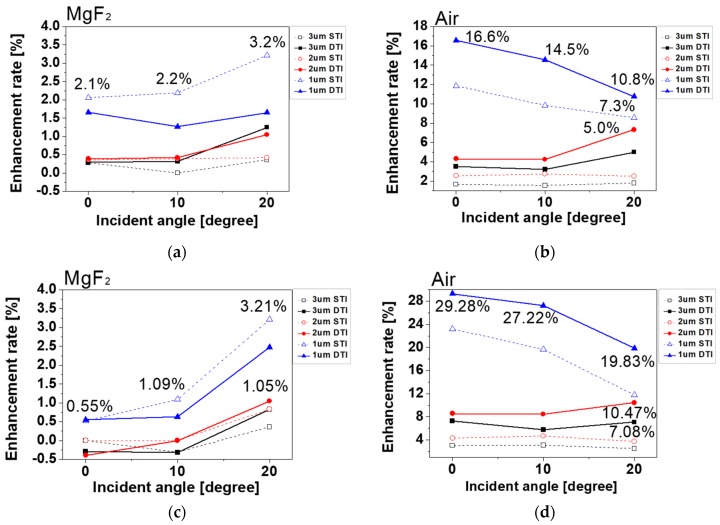
Simulation results of the enhancement rate incident angles of 0°, 10°, and 20°. Pixel depths were 1.0 μm (blue triangle symbol), 2.0 μm (red circle symbol), and 3.0 μm (black square symbol). The pixels with STI (dotted line) and DTI (solid line) were also compared: (**a**) Front-inner lens with MgF_2,_ (**b**) front-inner lens with air, (**c**) square type of front-inner lens with MgF_2_, and (**d**) square type of front-inner lens with air.

Finally, the absorbed photon density in the photodiode was simulated for investigation of the sensitivity as the pixel performance. Figure 4 shows the enhancement rate of the absorbed photon density between the typical and the suggested structures with respect to angular response. The enhancement rate was obtained by dividing the absorbed photon density of the typical and suggested structures. The simulated results according to the depth of the photodiode are represented by a blue triangle symbol for 1.0 μm, a red circle symbol for 2.0 μm, and a black square symbol for 3.0 μm. The simulation results of the pixels with STI are shown by a dotted line, and DTI are shown by a solid line. The simulation results of the enhancement rate are shown at the incident angles of 0°, 10°, and 20°. Figure 4a,b shows the enhancement rate in the case of the front-inner lens with MgF_2_ and air. Figure 4c,d shows the enhancement rate in the case of the square-shaped front-inner lens with MgF_2_ and air. From the simulation results, most pixels with the front-inner lens showed improvement in absorbed photon intensity. The 3.0 and 2.0 μm pixel depths did not exhibit such a large enhancement because the incident light was at 650 nm wavelength, which was absorbed completely at the 2.0 μm pixel depth. Therefore, the enhancement was greatest at 1.0 μm pixel depth. The enhancement rate in the case of air was larger than MgF_2_ because air, which has a lower refractive index and greater difference with the inter-dielectric layer, reflected the incident light more than the MgF_2_. The enhancement rate of the 1.0 μm pixel depth with DTI was the largest in the air front-inner lens structure. The reason for this was that the crosstalk was reduced by DTI, and the absorption was increased to the photodiode. As a result, the absorbed photon density as the sensitivity, compared between the typical and the suggested pixels, was improved to a maximum of 7.27%, 10.47%, and 29.28% at 3.0, 2.0, and 1.0 μm pixel depths, respectively.

**Figure 5:**
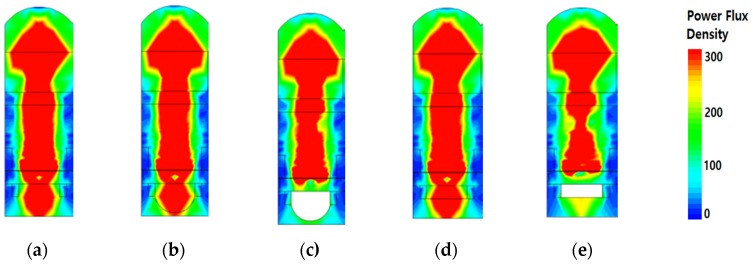
The beam profiles of the simulated power flux density. The incident light was a 650 nm wavelength at 0°. The mesh size was 5nm. The pixel depth was 1.0 μm with STI: (**a**) typical structure, (**b**) front-inner lens with MgF_2_, (**c**) front-inner lens with air, (**d**) square type of front-inner lens with MgF_2_ square, and (**e**) square type of the front-inner lens with air.

Figure 5 shows the beam profile of the power flux density when a 650 nm wavelength was incident at 0°. The simulated pixel depth was 1.0 μm and had an STI structure. Figure 5a shows that the incident light is passed from the micro lens to the photodiode and some lights are transmitted the front-side of a pixel. Figure 5b shows that the light was gathered in the front-inner lens because of the difference in the refractive index between MgF_2_ and the inter-dielectric layer. Moreover, the light was not transmitted and reflected to the photodiode, as shown in Figure 5c. Similarly, Figure 5d shows a front-inner lens using MgF_2_ in a square shape; there was not a large difference in beam profile between the square and the hemisphere shapes. Figure 5e shows a front-inner lens using air in a square shape: The light was not transmitted and reflected in the bottom of the photodiode. We can see that most of the light did not pass through and stayed in the photodiode by inserting the front-inner lens with air or air square.

**Figure 6:**
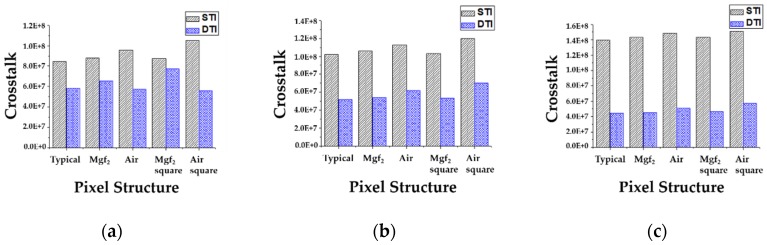
Simulation results of crosstalk at the incident angles of 0°, 10° and 20°. The pixel depths are 1.0 μm. The crosstalk in the pixels without DTI shows by the black diagonal stripe pattern, and the crosstalk with DTI shows by the blue check pattern. The pixels without the front-inner lens and with the front-inner lens of MgF2, MgF2 square type, air, and air square type are compared. (**a**) Crosstalk at the incident angles of 0°, (**b**) crosstalk at the incident angles of 10°, (**c**) crosstalk at the incident angles of 20°.

The crosstalk by the reflected light from the front-inner lens or the transmitted light from the next pixel can be reduced by the DTI, as mentioned in the Section 2. The simulation results of crosstalk are shown in Figure 6. Crosstalk is a sum of the absorbed light density of green and blue pixels when the incident light is 650 nm wavelength. We simulated the typical structure without the front-inner lens and the suggested structures with the front-inner lens of MgF2 or Air. The simulated pixel depth was 1.0 μm. Crosstalk is also investigated at the incident angles of 0°, 10°, and 20°. From the simulation results, crosstalk in the STI structure increased by inserting the front-inner lens. The reflected light was absorbed back into the pixel or penetrated into the next pixel to form a crosstalk. Also, the front-inner lens with air had more crosstalk because of its larger reflection. Therefore, the crosstalk should be reduced by DTI and the light can be absorbed into the pixel for high sensitivity. In Figure 6, the black diagonal stripe patterned bar indicated crosstalk without a DTI structure, and the blue check patterned bar indicates crosstalk with a DTI structure. For all of the structures, crosstalk can be enhanced by inserting DTI. As a result, crosstalk by DTI is reduced by up to 34%, 49%, and 68% at 0°, 10° and 20° incident angles, respectively. 

## 4. Conclusions

We introduced high-sensitivity pixels with the front-inner lens containing MgF_2_ or air. The front-inner lens was located in the interconnection layer in the backside illuminated sensor. The front-inner lens reflected the light of long wavelengths to absorb in the photodiode. Therefore, the pixel with the front-inner lens reduced the optical loss and increased sensitivity. Moreover, there was significant reflection by the air front-inner lens, where the refractive index of the front-inner lens was much lower than the inter-dielectric layer. The simulation results indicate that the front-inner lens structure can make the pixel depth shallower and provide higher sensitivity than is typical. Compared with the simulation results at depths of 1.0, 2.0, and 3.0 μm, the front-inner lens exhibited excellent performance at all pixel depths. In particular, at 1.0 μm pixel depth (the greatest improvement rate), we can see the possibility of a thin pixel structure. The enhancement was different at varying depths and was increased most prominently at 1.0 μm. From the results, we confirm the front-inner lens has good potential for the high-sensitive CMOS image sensor. The square-shaped front-inner lens with air type was the best inner-lens from the simulation result as it had the best enhancement rate of sensitivity at all angles in both the STI and DTI structures. 

For the future, the fabrication of the front-inner lens has to be considered in the backside illuminated (BSI) pixel. The front-inner lens can be fabricated between FEOL (front end of line) and BEOL (back end of line) processes. The FEOL process contains the trench (isolation) and well formation (n, p) for the photodiode, and the BEOL process indicates the metal wiring layer process. However, some difficulties for the process can be predicted. At first, the layout for the metal wiring layer is more complex because the front-inner lens should be located at the center of a pixel. Therefore, the layout has to be drawn to avoid the front-inner lens. Next, the lens shape formation can be affected the process of wiring layer such as the wiring pattern formation, interlayer insulating film deposition, the wiring contact or via formation. Since the wiring layer is processed after the lens is formed, the lens shape can crumble. Therefore, we need to research the fabrication method of the front-inner lens.

## Figures and Tables

**Table 1:** Simulated conditions.

**Pixel structure**	Pixel pitch	1.0 µm
	Pixel depths	3.0, 2.0, 1.0 µm
	Height & ROC of micro lens	0.6 µm
	Height & ROC of front-inner lens	0.25 µm/0.3 µm
	Width of WG	0.075 µm
	Height of WG	0.2 µm
	width of STI, DTI	0.1 µm
	Height of STI	0.35 µm
	DTI depths	3.0, 2.0, 1.0 µm
**Materials**	Refractive index of MgF_2_ & Air	1.3767 (650 nm)/1.0 (650 nm)
**Incident light**	Wavelength	650 nm
	Incident angle	0°, 10°, 20°

* ROC: radius of curvature, * WG: Tungsten Grid, * DTI: deep-trench isolation, * STI: shallow trench isolation.
